# Serum miRNA-1 may serve as a promising noninvasive biomarker for predicting treatment response in breast cancer patients receiving neoadjuvant chemotherapy

**DOI:** 10.1186/s12885-024-12500-6

**Published:** 2024-07-02

**Authors:** Jing Peng, Yanping Lin, Xiaonan Sheng, Chenwei Yuan, Yan Wang, Wenjin Yin, Liheng Zhou, Jinsong Lu

**Affiliations:** Present Address: Department of Breast Surgery, Renji Hospital, School of Medicine, Shanghai Jiao Tong University, No.160 Pujian Road, Shanghai, 200127 China

**Keywords:** Breast cancer, miRNA-1, Serum biomarker, Neoadjuvant chemotherapy

## Abstract

**Background:**

MicroRNA-1 (miR-1) is a tumour suppressor that can inhibit cell proliferation and invasion in several cancer types. In addition, miR-1 was found to be associated with drug sensitivity. Circulating miRNAs have been proven to be potential biomarkers with predictive and prognostic value. However, studies of miR-1 expression in the serum of breast cancer (BC) patients are relatively scarce, especially in patients receiving neoadjuvant chemotherapy (NAC).

**Methods:**

Serum samples from 80 patients were collected before chemotherapy, and RT-PCR was performed to detect the serum expression of miR-1. The correlation between miR-1 expression in serum and clinicopathological factors, including pathological complete response (pCR), was analyzed by the chi-squared test and logistic regression. KEGG and GSEA analysis were also performed to determine the biological processes and signalling pathways involved.

**Results:**

The miR-1 high group included more patients who achieved a pCR than did the miR-1 low group (*p *< 0.001). Higher serum miR-1 levels showed a strong correlation with decreased ER (R = 0.368, *p* < 0.001) and PR (R = 0.238, *p* = 0.033) levels. The univariate model of miR-1 for predicting pCR achieved an AUC of 0.705 according to the ROC curve. According to the interaction analysis, miR-1 interacted with Ki67 to predict the NAC response. According to the Kaplan–Meier plot, a high serum miR-1 level was related to better disease-free survival (DFS) in the NAC cohort. KEGG analysis and GSEA results indicated that miR-1 may be related to the PPAR signalling pathway and glycolysis.

**Conclusions:**

In summary, our data suggested that miR-1 could be a potential biomarker for pCR and survival outcomes in patients with BC treated with NAC.

**Supplementary Information:**

The online version contains supplementary material available at 10.1186/s12885-024-12500-6.

## Introduction

Breast cancer (BC) is the most commonly diagnosed malignant tumour in women worldwide [1, 2]. For locally advanced patients with a large tumour burden, neoadjuvant chemotherapy (NAC) is used as a regular BC treatment procedure. NAC can provide surgical opportunities for these patients and assess patient responsiveness to treatment. BC is a heterogeneous disease, and individual responses to chemotherapy are highly variable. Patients who achieve a pathological complete response (pCR) after NAC treatment have a much better prognosis than patients without a pCR, but only approximately 15–30% of patients achieve a pCR after NAC treatment [3–6]. Therefore, new clinical and biological tools are currently being developed to better predict patient responses to NAC and to individualize therapeutic strategies.

MicroRNAs (miRNAs) are a class of evolutionarily conserved noncoding RNAs that influence gene expression at the posttranscriptional level [7, 8]. Recent studies have identified circulating miRNAs as novel promising biomarkers for various diseases, including human malignancies [9, 10]. Serum serves as one of the most reliable samples among different body fluids. Extensive research has suggested that cancer‐related miRNAs can remain stable in serum under various conditions [11–13]. Therefore, a new serum miRNA marker for predicting and monitoring treatment efficacy is valuable and important for BC patients receiving NAC.

MiR-1 has been proven to be downregulated in some cancer types and to inhibit tumour growth [14–17]. Our previous research showed that miR-1 is expressed at low levels in BC tissues and that overexpression of miR-1 inhibits cell proliferation and migration [18]. An increasing number of serum miRNAs have been identified as important diagnostic or prognostic markers for BC [19–21]. However, it remains unknown whether miR-1 expression in serum can serve as a potential biomarker to predict the response to NAC in BC patients.

In this study, we aimed to explore the correlation between serum miR-1 expression and NAC sensitivity in patients in our weekly paclitaxel- and cisplatin-based neoadjuvant trial in BC patients. We hypothesized that high expression of miR-1 in the serum would be correlated with a good pCR and survival outcome for BC patients.

## Results

### Clinicopathological characteristics of the patients in the different miR-1 expression groups

MiR-1 was successfully detected in 80 patient serum samples. All of the patients were separated into 2 groups according to the median miR-1 expression level, and the clinicopathological baseline data of the two groups are shown in Table 1 and Supplementary Table 1. In the miR-1 high-expression group, ER expression was lower than that in the miR-1 low-expression group (Supplementary Table 1, *p* = 0.02). The pCR rate of NAC differed between the two groups. The miR-1 high group (62.5%) had more pCR patients (Table 1**,** *p* < 0.001) than did the miR-1 low group (22.5%), while other characteristics showed no obvious differences between the two groups. The specific miR-1 expression level of each sample and their pCR status are shown in Supplementary Fig. 1.

**Table 1:** Relationship between miR-1 expression and clinicopathological factors

Characteristics		miR-1 high expression (*n* = 40)		miR-1 low expression (*n* = 40)		*p* value*
		n	%	n	%	
Age	> 50	26	65.0%	26	65.0%	1
	≤ 50	14	35.0%	14	35.0%	
Post-menopause	Yes	23	57.5%	27	67.5%	0.356
	No	17	42.5%	13	32.5%	
ER	Positive	24	60.0%	29	72.5%	0.237
	Negative	16	40.0%	11	27.5%	
PR	Positive	30	75.0%	33	82.5%	0.412
	Negative	10	25.0%	7	17.5%	
HER2	Positive	33	82.5%	29	72.5%	0.284
	Negative	7	17.5%	11	27.5%	
Ki67	> 40%	21	52.5%	17	42.5%	0.37
	≤ 40%	19	47.5%	23	57.5%	
Tumor size	≤ 5 cm	10	25.0%	8	20.0%	0.592
	> 5 cm	30	75.0%	32	80.0%	
Lymph node metastasis	Positive	35	87.5%	32	80.0%	0.363
	Negative	5	12.5%	8	20.0%	
Histologic grade	I-II	14	35.0%	15	37.5%	0.816
	III	26	65.0%	25	62.5%	
NAC response	pCR	25	62.5%	9	22.5%	** < ** ***0.001***
	non-pCR	15	37.5%	31	77.5%	
* Pearson chi-square test						

*ER* Estrogen receptor, *PR* Progesterone receptor, *HER2* Human epidermal growth factor receptor 2,

*NAC* Neoadjuvant chemotherapy, *pCR* Pathological complete response

### Serum miR-1 is correlated with clinicopathological factors in patients with breast *cancer*

**Fig. 1:**
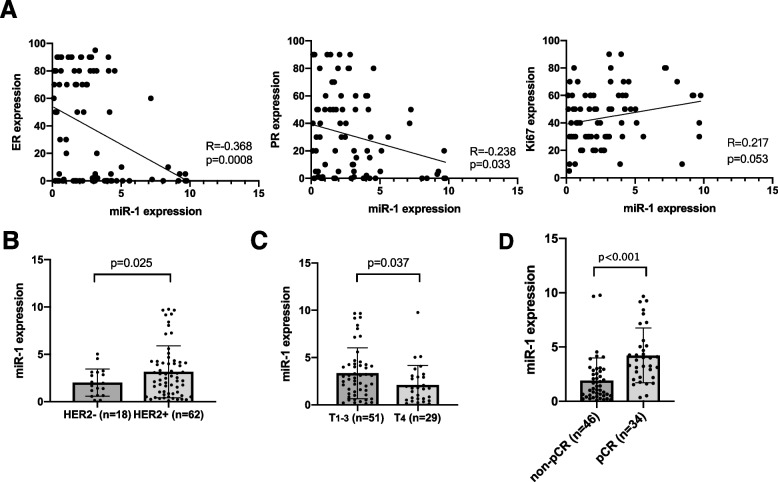
Expression levels of miR-1 and other breast cancer clinicopathological factors. **A** Correlations between miR-1 expression and the pathological factors ER, PR, and Ki67. **B** Differential serum miR-1 expression in the HER2-positive and HER2-negative subgroups. **C** Differential serum miR-1 expression in T4 patients and T1-3 patients. **D** MiR-1 expression in different NAC response groups

The correlation between miR-1 and clinicopathological factors was analysed in the NAC setting. Higher serum miR-1 levels showed a strong relationship with decreased ER (*R* = -0.368, *P* = 0.0008) and PR (*R* = -0.238, *p* = 0.033) levels (Fig. 1A). Serum miR-1 levels had no relationship with Ki67 expression (Fig. 1A, *R* = 0.217, *p* = 0.053). HER2-negative samples expressed a lower level of serum miR-1 (*p* = 0.025, Fig. 1B). Although serum miR-1 expression is related to pathological biomarkers, miR-1 expression was lower in the T4 stage samples (including tumours of any size directly extending to the chest wall and/or to the skin, and inflammatory BC) than in the T1-3 stage samples (*p* = 0.037, Fig. 1C). Moreover, for patients with different NAC responses, the serum miR-1 level was significantly higher in the pCR group than in the nonpCR group (*p* < 0.001, Fig. 1D).

### Serum miR-1 can predict the response to NAC in patients with breast *cancer*

**Fig. 2:**
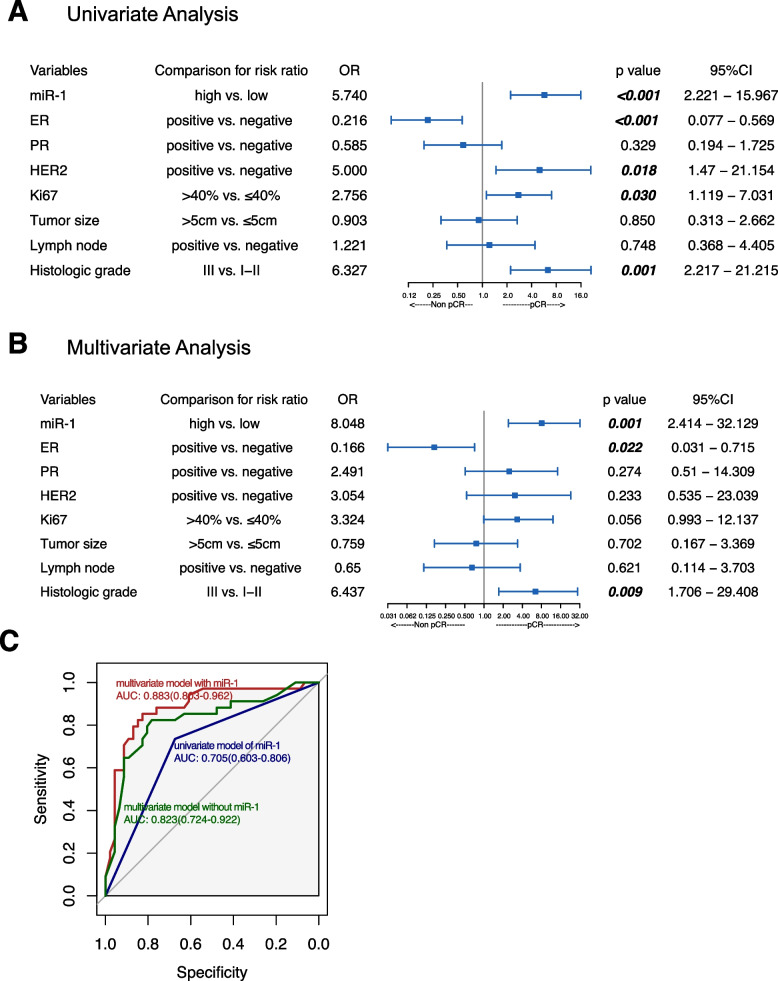
The value of miR-1 expression in predicting the response to neoadjuvant chemotherapy. **A** Univariate logistic regression analyses of potential predictive markers for predicting NAC response. **B** Multivariate logistic regression analyses of potential predictive markers for predicting NAC response. **C** ROC curves and AUCs were used to evaluate the performance of the models (a univariate model of miR-1, a multivariate model combining miR-1 and clinicopathological factors, and a multivariate model with only clinicopathological factors) in predicting NAC response

Logistic regression analysis was used to evaluate effective predictive markers of NAC response. First, univariate analysis of all patients indicated that serum miR-1 (OR = 5.740,* p *< 0.001, 95% CI 2.221–15.967), ER (OR = 0.216, *p* < 0.001, 95% CI 0.077–0.569), HER2 (OR = 5.0, *p* = 0.018, 95% CI 1.470–21.154), and Ki67 (OR = 2.756, *p* = 0.03, 95% CI 1.119–7.031) levels and the histologic grade (OR = 6.327, *p* = 0.001, 95% CI 2.217–21.215) were predictive markers of NAC pCR (Fig. 2A). According to the multivariate analysis, high miR-1 was also strongly related to a higher pCR rate of NAC (OR = 8.048, *p *= 0.001, 95% CI 2.414–32.129; Fig. 2B). The ER level (OR = 0.166, *p* = 0.022, 95% CI 0.031–0.715) and histological grade (OR = 6.437, *p* = 0.009, 95% CI 1.706–29.408) were also correlated with pCR according to the multivariate logistic regression (Fig. 2B). The predictive model of pCR based on serum miR-1 expression achieved an AUC of 0.705 according to the ROC curve (Fig. 2C). Moreover, a predictive model of pCR combining miR-1 and clinicopathological factors had a higher AUC than that of a predictive model with only clinicopathological factors (Fig. 2C).

### Predicting NAC response with serum miR-1 in different subgroups

**Fig. 3:**
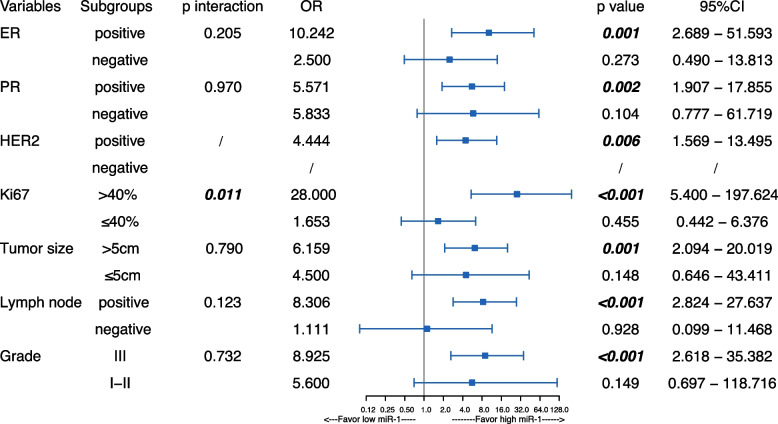
Value of serum miR-1 for predicting the response to neoadjuvant chemotherapy in different subgroups. Due to the low rate of pCR in the HER2- group, the odds ratio of miR-1 for predicting NAC response was not determined by logistic regression

Given that serum miR-1 levels could significantly predict the NAC response in the whole NAC cohort in our study, we further explored the predictive value of miR-1 in different subgroups (Fig. 3). According to the interaction analysis, miR-1 interacted with Ki67 to predict the NAC response (*p* = 0.011, Fig. 3). Specifically, miR-1 showed significant predictive value in the ER-positive group (OR = 10.242, *p* = 0.001, 95% CI 2.689–51.593), PR-positive group (OR = 5.571, *p* = 0.002, 95% CI 1.907–17.855), HER2-positive group (OR = 4.444, *p* = 0.006, 95% CI 1.569–13.495), high Ki67 group (OR = 28, *p* < 0.001, 95% CI 5.4–197.624), large tumour size group (OR = 6.159, *p* = 0.001, 95% CI 2.094–20.091), lymph node metastasis group (OR = 8.306, *p *< 0.001, 95% CI 2.824–27.637), and grade III group (OR = 8.925, *p* < 0.001, 95% CI 2.618–35.382). However, the predictive value of miR-1 in the other subgroups was not statistically significant (Fig. 3).

### Serum miR-1 is correlated with prognosis in the breast *cancer* NAC cohort

**Fig. 4:**
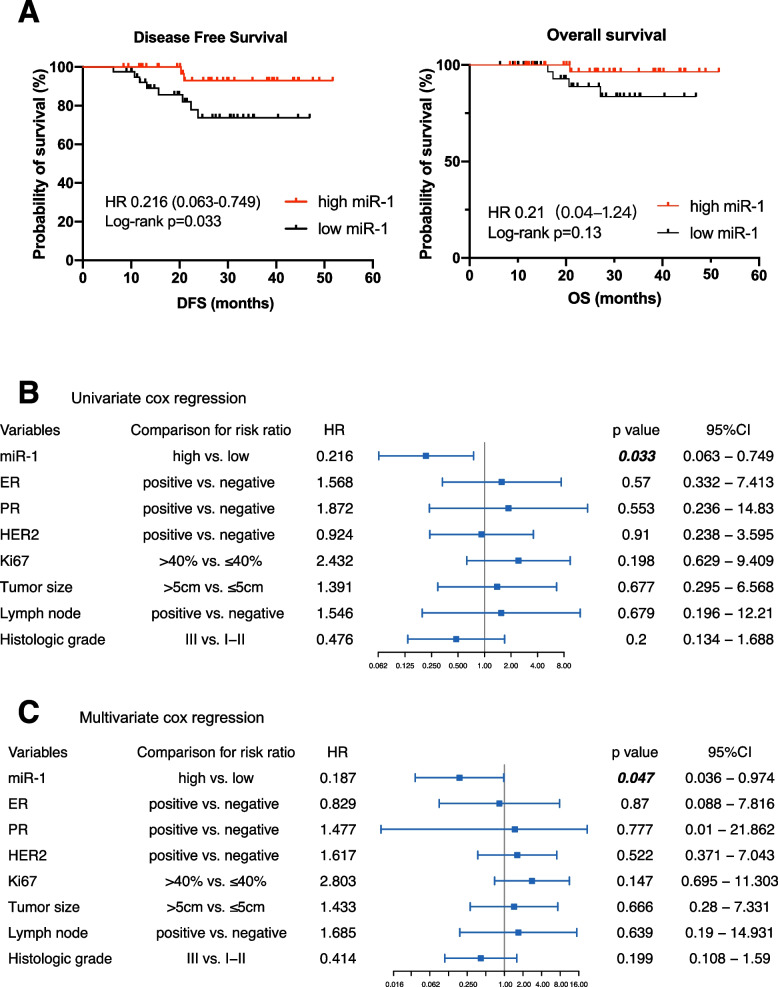
High serum miR-1 expression can predict a better prognosis in patients receiving neoadjuvant chemotherapy. **A **Kaplan-Meier plot of disease-free survival (DFS) and overall survival (OS) in the different miR-1 expression groups. **B** Univariate Cox regression analyses of potential predictive markers for NAC response. **C** Multivariate Cox regression analyses of potential predictive markers for NAC response

According to the Kaplan–Meier plot, a high serum miR-1 level was related to better DFS in the NAC cohort (HR 0.216, 95% CI 0.063–0.749; Fig. 4A). Overall survival (OS) was not significantly longer in the high-miR-1 group than in the low-miR-1 group (HR = 0.21, *p* = 0.13 and 95% CI 0.04–1.24; Fig. 4A). Univariate Cox regression revealed that miR-1 was the only factor significantly related to DFS (HR = 0.216, *p* = 0.033 and 95% CI 0.063–0.749; Fig. 4B). Multivariate analysis revealed that a high level of miR-1 also predicted better DFS (HR = 0.187 *p* = 0.047, 95% CI 0.036–0.974; Fig. 4C) in the NAC setting.

### Serum miR-1 participates in various *cancer*-related pathways

**Fig. 5:**
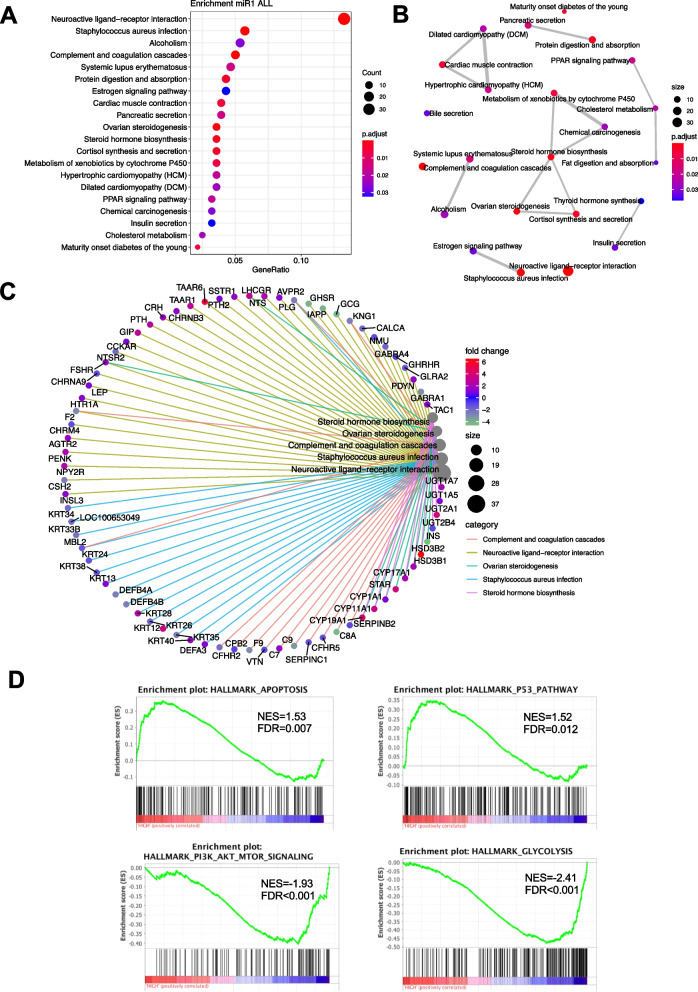
Differentially expressed genes (DEGs) in the different miR-1 expression groups from TCGA breast cancer dataset are enriched in various tumorigenesis- and chemosensitivity-related pathways. **A **Dot plot of KEGG pathway enrichment of DEGs in the different miR-1 expression groups. **B **Map plot of KEGG pathway enrichment of DEGs in the different miR-1 expression groups. **C** Cnetplot of KEGG pathway enrichment of DEGs in the different miR-1 expression groups. **D** Gene set enrichment analysis (GSEA) of DEGs in the different miR-1 expression groups

The TCGA dataset was separated into a miR-1 high group and a miR-1 low group, and differentially expressed genes (DEGs) were screened in the two groups. With thresholds of |logFC|> 1 and FDR < 0.05, 841 genes were upregulated, and 983 genes were downregulated in the miR-1 high group. KEGGpathway enrichment analysis indicated that miR-1 may be related to hormone activity, including the eestrogen signalling pathway, steroid hormone biosynthesis, ovarian steroidogenesis, and cholesterol metabolism (Fig. 5A-5C). DEGs were also enriched in other pathways involved in tumorigenesis or chemotherapy activity, such as the metabolism of cytochrome P450, the peroxisome proliferator-activated receptor (PPAR) signalling pathway, and chemical carcinogenesis (Fig. 5A-5C). GSEA revealed that miR-1 can upregulate apoptosis and the P53 pathway and downregulate PI3K-AKT-mTOR signalling and glycolysis (Fig. 5D).

## Discussion

Increasing evidence suggests that deregulated miRNA expression levels in cancer patients are associated with therapeutic outcomes. Previous studies have identified several predictors for treatment response to NAC, such as miR-21, miR-451, and miR-222, in BC [22–24]. In the present study, the expression level of serum miR-1 was shown to be a potential candidate biomarker for predicting the response to NAC and the prognosis of BC patients. This is the first study to analyze the expression level of circulating miR-1 in BC patients undergoing NAC and its relationship to treatment response.

NAC plays an important role in the treatment of locally advanced BC. Our previous study revealed that NAC combined with weekly paclitaxel and cisplatin was highly effective. The rate of pCR in the breast was 44.3%, and the rate of near-pCR in the breast was 48.1% [25]. The purpose of the present study was to identify new potential biomarkers that can predict the NAC response. Circulating miRNAs are more stable and more easily detected than tissue-based miRNAs and can be good biomarkers for predicting patients’ chemotherapeutic response [26–29]. MiR-1 was downregulated in the plasma of patients with oesophageal adenocarcinoma [30]. However, the relationship between serum miR-1 and the NAC response in BC patients has not been studied. The results showed that patients with high miR-1 levels achieved higher pCR rates than patients with low miR-1 expression levels and that miR-1 could be an independent predictive biomarker for NAC sensitivity in BC patients. Consistent with our results, Hua revealed that miR-1 expression was downregulated in cisplatin-resistant non-small cell lung cancer (NSCLC) patients and that miR-1 overexpression improved the cisplatin sensitivity of NSCLC cells [31]. Yu demonstrated that a high level of miR-1 could increase the sensitivity of oesophageal squamous cell carcinoma (ESCC) cells to the anticancer drug gefitinib [32]. Deng revealed that miR-1 reverses multidrug resistance in gastric cancer cells by promoting the accumulation of intracellular drugs [33].

pCR in the primary tumour following NAC is a strong predictor of long-term survival. We next evaluated the potential value of serum miR-1 as a candidate marker to predict the postoperative prognosis of BC patients and found that low serum miR-1 was significantly related to poor survival in BC patients. The same phenomenon was observed in other tumours, and serum miR-1 was also identified as having a significant correlation with progression-free survival (PFS) in advanced NSCLC patients and functions as a protective factor [34]. Waidmann reported that hepatocellular carcinoma patients with higher serum miR-1 levels had a longer OS than those with lower miR-1 levels [35]. Liu revealed that miR-1 expression in plasma was closely related to invasion and lymph node metastasis in ESCC [14]. To the best of our knowledge, our study is the first to identify serum miR-1 as a novel predictive circulating biomarker of the efficacy of NAC for BC. Therefore, miR-1 expression in serum may serve as a novel potential biomarker for NAC chemosensitivity and a novel prognostic biomarker for BC.

Interestingly, we found that the serum miR-1 level showed a strong negative correlation with ER level. In addition, TargetScan showed that ER could be a direct target of miR-1, suggesting that miR-1 can downregulate ER expression. Furthermore, miR-1 showed an interaction with Ki67 to predict the NAC response in our study. Ki67 is a marker of cell proliferation, and the interaction between miR-1 and Ki67 therefore indirectly indicated that miR-1 is related to cell proliferation. Despite promoting chemotherapy sensitivity, evidence also suggests that miR-1 inhibits cell proliferation, including epithelial cells and cancer cells [36, 37]. Based on the interaction of miR-1 and Ki67, we noticed significant enhancement of the chemotherapy response with high miR-1 in the high Ki67 group, reflecting a remarkable role for miR-1 in increasing chemosensitivity in subgroups with high proliferation potential. The expression of these miRNAs in BC and their correlation with ER/PR/HER2/Ki67 may further elucidate the pathogenic mechanisms of these well-known receptors in BC development.

Our results showed that miR-1 upregulation might lead to increased sensitivity to NAC. However, the underlying mechanism remains unknown. Yang revealed that miR-1 regulates the growth and chemosensitivity of BC cells by targeting the MEK/ERK pathway [38]. The MEK/ERK pathway plays pivotal roles in chemotherapeutic drug resistance [39, 40]. McCubrey reported that ERK is activated after tamoxifen and doxorubicin treatment in MCF-7 BC cells [41]. Furthermore, drug-resistant cells exhibit increased levels of constitutively active ERK. Many specific MEK inhibitors have been developed and may increase the sensitivity of cells to chemotherapies, hormonal agents, and immunotherapies [42–44]. Wu reported that miR-1 can affect BC stem cell proliferation. Stem cells are regarded as the key factor for chemotherapy resistance in BC due to their high tumorigenicity and metastatic potential [17]. Tao demonstrated that miR-1 upregulation in cancer-associated fibroblast (CAF)-derived extracellular vesicles (EVs) successfully impaired BC progression and metastasis. CAFs play supporting roles in tumour progression by releasing microvesicles that transmit oncogenic cargoes. EVs can help to deliver proteins, mRNAs, and miRNAs between cells. EV-carried miRNAs are recognized as diagnostic markers of BC and have the potential to serve as promising therapeutic targets [45].

KEGG analysis revealed that miR-1 may be related to hormone activity, the metabolism of cytochrome P450, and the PPAR signalling pathway, which all play important roles in BC tumorigenesis and chemotherapy [46]. The cytochrome P450 (CYP) family is a multigene family of enzymes that play key roles in the metabolism of a diverse range of drugs. CYP polymorphisms are associated with NAC efficacy in BC patients. For example, in CYP2C9*2 heterozygotes with a high hereditary load, the risk of resistance to NAC was much higher than that in wild-type homozygotes. In addition, rs17102977 in CYP4X1 is significantly associated with DFS in NAC patients [47, 48]. The PPAR pathway plays a pivotal role in the response to chemotherapy. Chen identified a number of genes pertinent to the therapeutic response in BC patients using a weekly paclitaxel plus carboplatin regimen and found that the PPAR signalling pathway may be a potential predictor of the response of BC patients to NAC [49]. Thus, the potential interaction between miR-1 and PPARs might be crucial in the development of resistance to chemotherapeutic drugs.

GSEA revealed that miR-1 may upregulate apoptosis and the p53 pathway, which could suppress cancer development. p53 is a well-known tumour suppressor and plays an important role in the response to DNA damage and oncogenic signalling [50]. In patients who received NAC, p53 expression was associated with better BC-specific survival rates in patients without lymph node metastasis. A study also revealed that p53-positive BC patients receiving NAC had a better OS than p53-negative BC patients receiving NAC [51]. Furthermore, we found that miR-1 can also downregulate PI3K-AKT-mTOR signalling and glycolysis pathways. Similarly, Deng revealed that overexpression of miR-1 suppressed gastric cancer cell proliferation by inhibiting aerobic glycolysis [52]. Aerobic glycolysis is a characteristic tumour cell phenotype that can promote tumour progression by accelerating glucose uptake and lactate production. Groheux reported that total lesion glycolysis may be an early predictor of tumour regression and treatment outcomes after NAC. Lactate dehydrogenase A (LDHA) and LDHB are lactate dehydrogenase tetrameric enzymes utilized by cancer cells to produce lactate from pyruvate. Dennison found that LDHB was highly expressed in aggressive, glycolytic BC, primarily of the basal subtype, and could predict the response to NAC [53, 54]. In line with our results, miR-1 was found to be related to androgen receptor activity and the PI3K signalling pathway in prostate cancer [55, 56]. The PI3K-AKT-mTOR signalling pathway is a key regulator of glycolysis and plays an important role in the growth and migration of cancer cells [57]. The PI3K-AKT-mTOR pathway is also correlated with resistance to chemotherapy. Some studies have suggested that elevated AKT expression is associated with a poor prognosis in BC. Therefore, many clinical trials have targeted the PI3K-AKT-mTOR pathway. The expression level of p-MTOR may be a more reliable predictor of DFS in NAC patients [58, 59]; therefore, it is important to determine the mechanism of miR-1 in chemosensitivity in future studies, and in vitro and in vivo experiments are needed to validate these hypotheses.

A notable limitation of our study is that it was conducted at a single centre, and the sample size was small. A large-scale, multicentre prospective study with a longer follow-up is needed to validate our results. Furthermore, in vitro and in vivo experiments need to be conducted to reveal the specific molecular pathway by which miR-1 regulates chemosensitivity in BC. Another limitation is that in this study, only baseline serum levels of miR-1 were evaluated, so further research to explore the serum levels of miR-1 in different phases of NAC is needed in the future.

In conclusion, our study revealed that serum miR-1 is a potential factor for predicting pCR and survival benefits in BC patients receiving NAC.

## Materials and methods

### Patient cohort and treatment

All enrolled BC patients were consecutively included from two paclitaxel- and cisplatin-based neoadjuvant clinical trials. The two trials were registered as SHPD001 (NCT02199418, 14/07/2014) and SHPD002 (NCT02221999, 14/07/2014) at ClinicalTrials.gov. The two trials were verified and approved by the Independent Ethical Committee of Renji Hospital, Shanghai Jiao Tong University. We confirm that all methods were performed in accordance with relevant guidelines and regulations. Women aged ≥ 18 and ≤ 70 years with locally advanced invasive BC were included in the two clinical trials. Oestrogen receptor (ER) and progesterone receptor (PR) status was defined as positive if more than 10% of the tumour cells exhibited positive staining by IHC. HER2 positivity was defined as IHC 3 + or fluorescence in situ hybridization (FISH) amplification. The participants were subjected to 4 cycles (28 days for each cycle) of paclitaxel 80 mg/m^2^ every day for 1, 8, 15, or 21 days in each cycle and cisplatin 25 mg/m^2^ for 1, 8, or 15 days every 28 days in each cycle. Dexamethasone was given intravenously before chemotherapy. HER2-positive patients were given trastuzumab on a weekly basis. Trastuzumab was given at 4 mg/kg body weight for the first dose and at 2 mg/kg for any subsequent doses. The pathological NAC response was evaluated according to the surgical samples. The additional treatment protocols used in the two trials have been reported previously [25, 60]. After completion of NAC, the patients underwent planned surgery. The primary outcome of the two trials was pCR, which was defined as the absence of invasive tumours in the breast and axillary lymph node samples. DFS was defined as the time from surgery after neoadjuvant chemotherapy to the first time of locoregional recurrence, ipsilateral/contralateral recurrence, distant recurrence or death from any cause.

### Extraction of total RNA and reverse transcriptase-quantitative polymerase chain reaction (RT-qPCR)

For each patient, total RNA from 300 μl serum was isolated using the mirVana PARIS kit (Ambion, Texas, United States) according to the manufacturer’s instructions. cDNA was synthesized by reverse transcription of total RNA with the TaqMan MicroRNA Reverse Transcription Kit (Ambion, Texas, United States). After that, 100 ng of total RNA was reverse-transcribed using specific primers. The TaqMan primers used for hsa-miR-1 and cel-miR-39 were obtained from Applied Biosystems (RT002222 and RT000200). For RT-qPCR, miRNA-specific TaqMan Small RNA Assays (Ambion, Texas, United States) for hsa-miR-1 and cel-miR-39 were used as described by the manufacturer. cel-miR-39 was used as an endogenous control to normalize the serum samples. Real-time PCR was performed on a LightCycler® 480 II (Roche, Mannheim, Germany) system using a probe qPCR kit (RR390A, TaKaRa, Dalian, China). The expression level of hsa-miR-1 relative to that of cel-miR-39 was calculated using the 2^−ΔCT^ method, where ΔCt = mean value Ct (miR-1)-mean value Ct (reference miR-39). All reactions were performed in triplicate.

### Statistical analysis

Serum miR-1 expression was separated into high or low groups based on the median level of serum miR-1 expression in the NAC setting. Univariate and multivariate logistic regression analyses were performed to evaluate the association between serum miR-1 expression and the NAC response, with parameters including odds ratios (ORs), 95% confidence intervals (95% CIs), and p values. A Kaplan–Meier plot was generated for the survival analysis of different serum miR-1 expression groups. Univariate and multivariate Cox regression models were used to screen for factors related to the prognosis of patients receiving NAC, with hazard ratios (HRs), 95% confidence intervals (95% CIs), and p values. Receiver operating characteristic (ROC) curves and areas under the curve (AUCs) were used to verify the performance of the prediction models. Classified variables were compared by the chi-square test, while the continuous variables were compared by the Student’s t test. Correlations between miR-1 expression and the continuous variables ER, PR, and Ki67 were analyzed by Pearson correlation analysis.

Statistical analyses were performed with R (version 3.4.3) and SPSS (version 23.0.0.0). A two-sided P value < 0.05 was considered to indicate statistical significance. The figures were drawn and edited in GraphPad Prism 8, R (version 3.4.3), and Adobe Illustrator (version 21.0.0.0).

### Bioinformatic analysis

The expression of miR-1 and mRNA in TCGA BC dataset was downloaded from https://portal.gdc.cancer.gov. Differentially expressed genes (DEGs) were screened with the R package “edgeR”. Kyoto Encyclopedia of Genes and Genomes (KEGG) pathway enrichment analysis was performed with the R package “enrichplot”. Gene set enrichment analysis (GSEA) was performed with GSEA 4.0.1 software.

### Supplementary Information

Supplementary Material 1: Supplementary Figure 1. miR-1 expression level and pCR status of each sample.

Supplementary Material 2: Supplementary Table 1. Clinicopathological baseline of different miR-1 expression groups.

## Data Availability

The authors confirm that the data supporting the findings of this study are available within the article and its supplementary materials.
