# Supplementary figures and images for: Serum miRNA-1 may serve as a promising noninvasive biomarker for predicting treatment response in breast cancer patients receiving neoadjuvant chemotherapy
Source: BMC Cancer. 2024 Jul 2;24:789. doi: 10.1186/s12885-024-12500-6 (PMC11221026; doi:10.1186/s12885-024-12500-6)

miR1 expression

non-pCR  
pCR

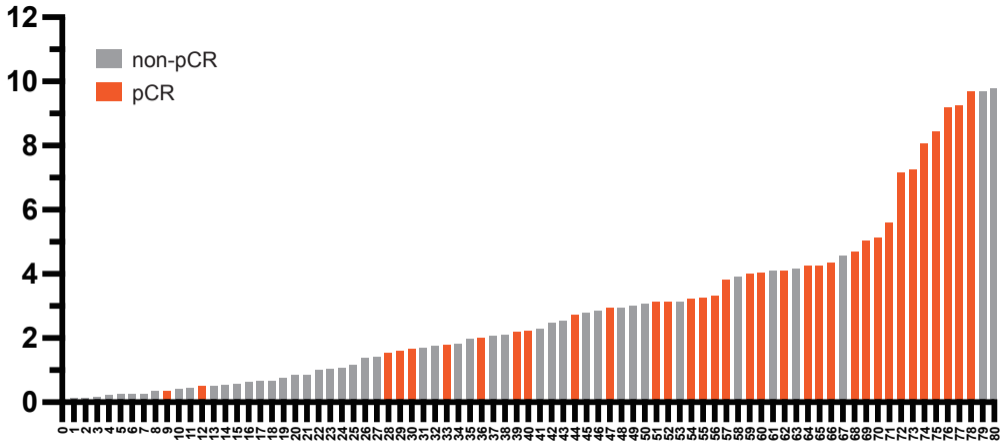

Supplement: Supplementary file 1 — Supplementary Material 1: Supplementary Figure 1. miR-1 expression level and pCR status of each sample. [file 12885_2024_12500_MOESM1_ESM.pdf]
