# Supplementary material for: Serum miRNA-1 may serve as a promising noninvasive biomarker for predicting treatment response in breast cancer patients receiving neoadjuvant chemotherapy
Source: BMC Cancer. 2024 Jul 2;24:789. doi: 10.1186/s12885-024-12500-6 (PMC11221026; doi:10.1186/s12885-024-12500-6)
Supplement: Supplementary file 2 — Supplementary Material 2: Supplementary Table 1. Clinicopathological baseline of different miR-1 expression groups. [file 12885_2024_12500_MOESM2_ESM.docx]

Supplementary Table 1. Clinicopathological baseline of different miR1 expression groups

| Characteristics | miR1 high expression (n=40)  median (95%CI, min, max) | miR1 low expression (n=40)  median (95%CI, min, max) | P |
| --- | --- | --- | --- |
| miR1 expression | 4.05(3.08-5.478, 2.28, 9.77) | 0.86(0.40-1.68, 0.11, 2.2) | <0.001 |
| Age (year) | 54.5(45-61, 26, 70) | 53(47-58, 25, 71) | 0.98 |
| ER (%) | 5(0-70, 0, 95) | 65(0-80, 0, 90) | 0.02 |
| PR (%) | 17.5(0.-50, 0, 90) | 35(2-60, 0.90) | 0.1 |
| Ki67 (%) | 50(30-60, 10, 90) | 40(30-57, 5, 80) | 0.1 |
| Tumor size (cm) | 7(5-9, 2, 12) | 7.5(5.8-9.3, 0, 16) | 0.2 |
| Height (cm) | 160(157.3-165, 150, 171) | 160(156-160.8, 148, 176) | 0.07 |
| Weight (kg) | 58(52-65, 42.5,87) | 58.8(55-64.8, 40, 81) | 0.8 |
